# Methylation-sensitive restriction endonuclease-based amplification assay for detecting DNA methylation in colorectal cancer: a cross-sectional study

**DOI:** 10.1186/s12876-026-05034-7

**Published:** 2026-08-03

**Authors:** Jiali Wu, Zhikang Li, Lu Ai, Anzhi Huang, Shayuanzi Huang, Ruizhao Zhang, Guoshan He, Meifang He, Ruizhi Wang, Dong Wang

**Affiliations:** 1 Department of Laboratory Medicine, The First Affiliated Hospital of Sun Yat-sen University, 58 Zhongshan road II, Guangzhou, Guangdong China; 2 Laboratory of General Surgery, The First Affiliated Hospital of Sun Yat-Sen University, 58 Zhongshan road II, Guangzhou, Guangdong China; 3 Guangdong Cardiovascular Institute, Medical Research Institute, School of Basic Medical Science, Guangdong Provincial People’s Hospital, Guangzhou, Guangdong China; 4 Department of Laboratory Medicine, Guangxi Hospital Division of The First Affiliated Hospital of Sun Yat-sen University, Nanning, Guangxi China; 5 Guangzhou Quality Testing & Inspection Institute, Guangzhou, Guangdong China; 6 Advanced Medical Technology Center, The First Affiliated Hospital, Zhongshan School of Medicine, Sun Yat-Sen University, Guangzhou, Guangdong China; 7 Guangdong Provincial Clinical Research Center for Laboratory Medicine, Guangzhou, P. R. China

**Keywords:** Methylation-sensitive restriction endonuclease, ADHFE1, SDC2, Colorectal cancer, Methylation

## Abstract

**Background:**

This study evaluates the clinical utility of combined SDC2 and ADHFE1 methylation analysis for colorectal cancer (CRC) detection using a methylation-sensitive restriction endonuclease (MSRE)-based assay.

**Methods:**

Leveraging the specificity of MSRE, we developed a streamlined amplification assay that directly analyzes methylation status of SDC2 and ADHFE1 after mixing nucleic acids with PCR reagent. A cohort of 274 participants was stratified into six groups: CRC (*n* = 117), advanced adenoma (AA, *n* = 22), polyps (*n* = 30), intestinal disease control (IDC, *n* = 39), other gastrointestinal disease control (OGDC, *n* = 24), and healthy controls (HC, *n* = 42). SDC2 and ADHFE1 methylation levels in fecal samples were assessed, and diagnostic performance was evaluated using receiver operating characteristic (ROC) curve analysis.

**Results:**

The CRC group exhibited significantly higher SDC2 and ADHFE1 methylation positivity rates compared to all comparator groups, including AA, polyps, IDC, OGDC, and HC (*P* < 0.01). No significant differences were observed in the methylation positivity rates of SDC2, ADHFE1 or their combination across different TNM stages and levels of tumor differentiation. Joint SDC2 and ADHFE1 analysis increased sensitivity for CRC detection to 82.9% (vs. 64.1% and 65.0% for ADHFE1 and SDC2) with an AUC of 0.865 (95% CI: 0.817–0.913). For early-stage CRC (stages I + II), the dual-gene panel achieved an AUC of 0.872. However, the ability to discriminate between AA and HC (AUC: 0.693) or IDC (AUC: 0.615) remained limited in our dual-gene assay.

**Conclusions:**

The combined SDC2/ADHFE1 MSRE-based assay significantly improves diagnostic sensitivity for CRC, demonstrating superior differentiation of CRC from benign intestinal pathologies and precancerous lesions (AA and polyps). This non-invasive approach shows promise as a supplementary tool for CRC screening.

**Supplementary Information:**

The online version contains supplementary material available at https://doi.org/10.1186/s12876-026-05034-7.

## Introduction

Colorectal cancer (CRC) ranks as the third most common cancer globally and the second leading cause of cancer-related mortality [1]. In China, CRC incidence and mortality are rising, ranking second and fifth among all malignancies, respectively. While the overall 5-year survival rate for CRC remains suboptimal at approximately 60% [2], it approaches 90% for localized cases but plummets to just 14% for metastatic disease [3]. This dramatic disparity underscores the critical importance of early diagnosis for improving prognosis [4]. The adenoma-carcinoma sequence, driven by genetic and epigenetic alterations [5, 6] provides a window for early intervention during the 5–10-year progression from precancerous lesions to invasive carcinoma [5].

Colonoscopy remains the gold standard for CRC screening but faces limitations due to invasiveness, cost, and accessibility [7]. Non-invasive alternatives such as the fecal occult blood test (FOBT) and fecal immunochemical test (FIT) exhibit suboptimal sensitivity for detecting AA [8]. Aberrant DNA methylation, an early event in carcinogenesis, offers promise as a biomarker for early non-invasive screening [9, 10].

SDC2 and ADHFE1 are hypermethylated in CRC and have been strongly implicated in CRC pathogenesis. Fecal SDC2 methylation detection achieves 77.4% sensitivity for CRC [11], while hypermethylation of ADHFE1 that regulates 4-hydroxybutyrate metabolism in mammalian tissues [12], correlates with poor CRC differentiation and advanced stage [13]. Multi-gene panels may enhance diagnostic sensitivity compared to single-marker assays, particularly for early-stage CRC detection [14–17]. For example, combined SDC2/ADHFE1/PPP2R5C methylation analysis reached 84.8% sensitivity and 98.0% specificity for detecting CRC [18].

Current methylation detection primarily employs bisulfite conversion, a process characterized by harsh reaction conditions and technically demanding procedures. Furthermore, post-conversion purification requirements introduce risks of DNA loss, potentially compromising the accuracy of downstream analyses. In contrast, MSRE-based assays precisely discriminate methylation from unmethylated DNA by identifying the recognition site and selectively cleaving unmethylated target sequences, leaving methylated DNA intact [17]. This approach preserves nucleic acid integrity and enhances detection sensitivity by eliminating bisulfite conversion requirements. Moreover, combining enzymatic treatment with amplification in a single reaction vessel significantly simplifies experimental workflows.

By specifically recognizing and cleaving at target sites, MSRE digests unmethylated DNA into short fragments while leaving methylated DNA intact, thereby enabling effective differentiation and identification of methylated and unmethylated DNA. Leveraging the highly specific recognition and digestion capability of MSRE and the exponential amplification capacity of PCR, we have developed an MSRE-based assay for DNA methylation detection. HpaII and HhaI specifically cleave unmethylated targets, while leaving methylated targets intact. Subsequently, the intact methylated target is capable of initiating a subsequent cascade exponential amplification reaction, whereas the cleaved unmethylated target fails to trigger further amplification.

In the present study, we employed an enzyme mixture of HpaII and HhaI to maximize CpG site coverage while minimizing false negatives in epigenetically resistant regions. We further integrated MSRE digestion and PCR into a single-tube workflow to enable rapid, sensitive detection of SDC2 and ADHFE1 methylation in fecal DNA and further evaluate its diagnostic accuracy for CRC screening.

## Materials and methods

### Patients cohort

**Fig. 1:**
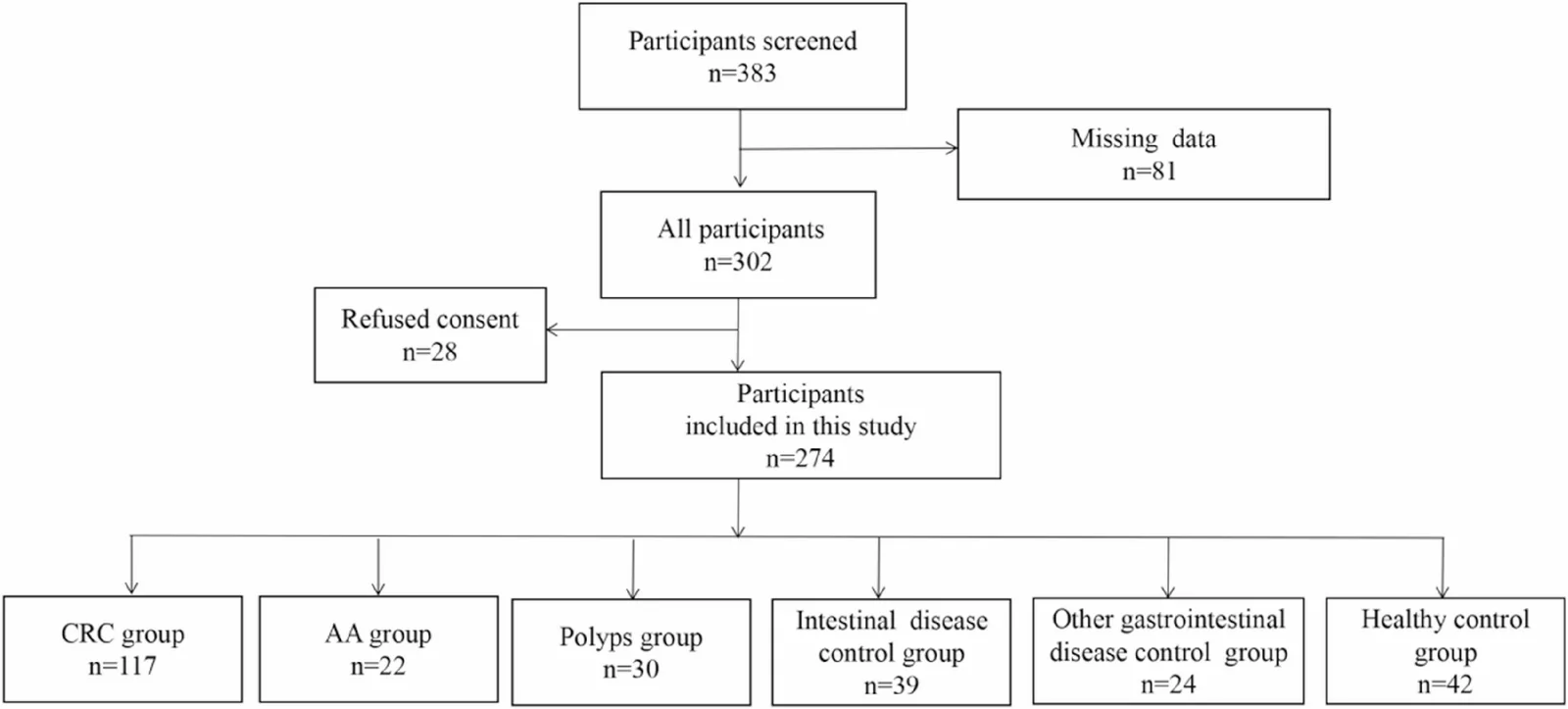
STROBE flow diagram of participant selection and grouping

This was a cross-sectional study. From July 2023 to January 2025, 274 participants who underwent colonoscopy at the First Affiliated Hospital of Sun Yat-sen University were enrolled. Based on colonoscopy and histopathology, participants were stratified into six groups including CRC (*n* = 117), AA (*n* = 22), polyps (*n* = 30), IDC (*n* = 39), OGDC (*n* = 24) and HC (*n* = 42) (Fig. 1). The sample size was determined based on the availability of eligible participants at our single center during the study period, rather than a formal a priori calculation. This cross-sectional study was approved by the Ethics Committee of the First Affiliated Hospital of Sun Yat-sen University (Approval No. [2024]755; Approval Date: 2025.01.23).

The study was conducted in accordance with the Declaration of Helsinki. Written informed consent was obtained from all participants. This study was designed and reported in accordance with the STROBE (Strengthening the Reporting of Observational Studies in Epidemiology) guidelines to ensure transparency and completeness in reporting observational research. 

### DNA isolation and methylation detection of SDC2 and ADHFE1 genes

Fecal DNA was co-extracted using a commercial kit (EasBead, YiXin Biotech, China) following the manufacturer’s protocol. For MSRE-based PCR, the enzyme mix was prepared as an equal-volume mixture of HpaII and HhaI (Thermo Scientific™ FD0514 and FD1854, respectively). The PCR was performed using predesigned probes and primers (Table 1) to assess methylation levels of SDC2 and ADHFE1, with β2-microglobulin (β2-M) amplification serving as an internal control, while ensuring the integrity of sample DNA to prevent degradation.

**Table 1:** Primers and probes

		Sequence 5′-3′
β2-M	forward primer	CTGGGTTTCATCCATCCGAC
	Reverse primer	ATCTTGGGCTGTGACAAAGT
	probe	ATGCCTGCCGTGTGAACCAT
SDC2	SDC2 forward primer	GGCGGAGTCGGTGAGT
	SDC2 reverse primer	GCGAGCAGCGCTTTTCT
	SDC2 probe	CCGGACGAGCGCATTTGTCC
ADHFE1	ADHFE1 forward primer	GCGTACTTGCTGAGGCAACT
	ADHFE1 reverse primer	CGGTATCCAAATTCTCCGAAT
	ADHFE1probe	CAGATCCTGCGCAGTCTTCTC

By specifically recognizing and cleaving at target sites, HpaII (recognizing the C/CGG sequences) and HhaI (recognizing the GCG/C site) digests unmethylated DNA into short fragments while leaving methylated DNA intact, thereby enabling effective differentiation and identification of methylated and unmethylated DNA. Subsequently, the intact methylated target is capable of initiating a subsequent cascade exponential amplification reaction, whereas the cleaved unmethylated target fails to trigger further amplification. Each PCR contains 25 µl of the reaction mixture, and the reaction system is shown in Table 2.The PCR were performed on the SLAN-96 S analyser according to the following thermal cycling conditions: 37 °C for 8 min, 95 °C for 1 min, 45 cycles of 95 °C for 15 s, 60 °C for 15s, 72 °C for 20 s. The ΔCt (ADHFE1-β2-M) value ≤ 10 was considered to be ADHFE1-positive and the ΔCt (SDC2-β2-M) value ≤ 9 was considered to be SDC2-positive. Undetected Ct values were considered to be SDC2/ADHFE1 negative.

**Table 2:** Reaction system

Reagent	Volume	Reagent	Volume
Restriction enzyme digested amplification enhanced buffer	12.5 µL	β2-M-F1 (100 µM)	0.02 µL
ADHFE1-F1 (100 µM)	0.08 µL	β2-M-R1 (100 µM)	0.02 µL
ADHFE1-R1 (100 µM)	0.08 µL	β2-M-P1 (100 µM)	0.01 µL
ADHFE1-P1 (100 µM)	0.04 µL	Taq DNA polymerase (5 U/µL)	0.5 µL
SDC2-F1 (100 µM)	0.04 µL	Methylation-sensitive restriction enzyme mix	0.1 µL
SDC2-R1 (100 µM)	0.04 µL	DNA template	5 µL
SDC2-P1 (100 µM)	0.02 µL	Nuclease-free water	6.55 µL

### Data sources and measurement

All clinical and demographic information of the participants, including age, gender, colonoscopy findings, and definitive histopathological diagnoses, was retrospectively extracted from the structured electronic medical record (EMR) system of the First Affiliated Hospital of Sun Yat-sen University. These records served as the gold standard for categorizing participants into disease groups (e.g., CRC and AA). The key biomarker data, namely the methylation status of the SDC2 and ADHFE1 genes in fecal samples, were generated using our laboratory-developed MSRE-PCR assay. The specific criteria for determining a positive methylation result strictly followed the manufacturer’s instructions of the assay kit, utilizing the ΔCt value (Ct of the target gene minus Ct of the reference gene β2-M) with predefined cut-off thresholds.

### Assessment and control of potential biases

To minimize selection bias, we aimed to consecutively enroll all eligible participants who underwent colonoscopy at our center during the study period. To reduce measurement error, laboratory personnel performing the MSRE-PCR assay and subsequent data analysis were blinded to the clinical diagnosis of all samples. Standardized commercial kits were used for DNA extraction, and predefined, objectively calculated ΔCt thresholds were applied to determine methylation positivity, thereby minimizing subjective interpretation. We recognized that factors like age and sex could confound the observed associations. In our analysis, we have provided both the unadjusted and adjusted (for age and sex) data.

### Statistical analysis

Categorical variables were expressed as frequency counts (percentages) and intergroup comparisons were performed using chi-square (χ²) tests or Fisher’s exact probability tests. The correlation between colorectal cancer (CRC) and the combined ADHFE1/SDC2 detection was assessed by calculating the odds ratio (OR) and 95% confidence interval (CI) using logistic regression models. The ROC curve analysis was used to assess the diagnostic effectiveness of SDC2 and ADHFE1 for CRC. Statistical analyses were conducted using SPSS (v26.0; IBM Corp., USA) and GraphPad Prism (v10.0; GraphPad Software Inc., USA), with statistical significance defined as a two-tailed *P*-value < 0.05.

## Results

### Validation of the MSRE-based DNA methylation detection assay

**Fig. 2:**
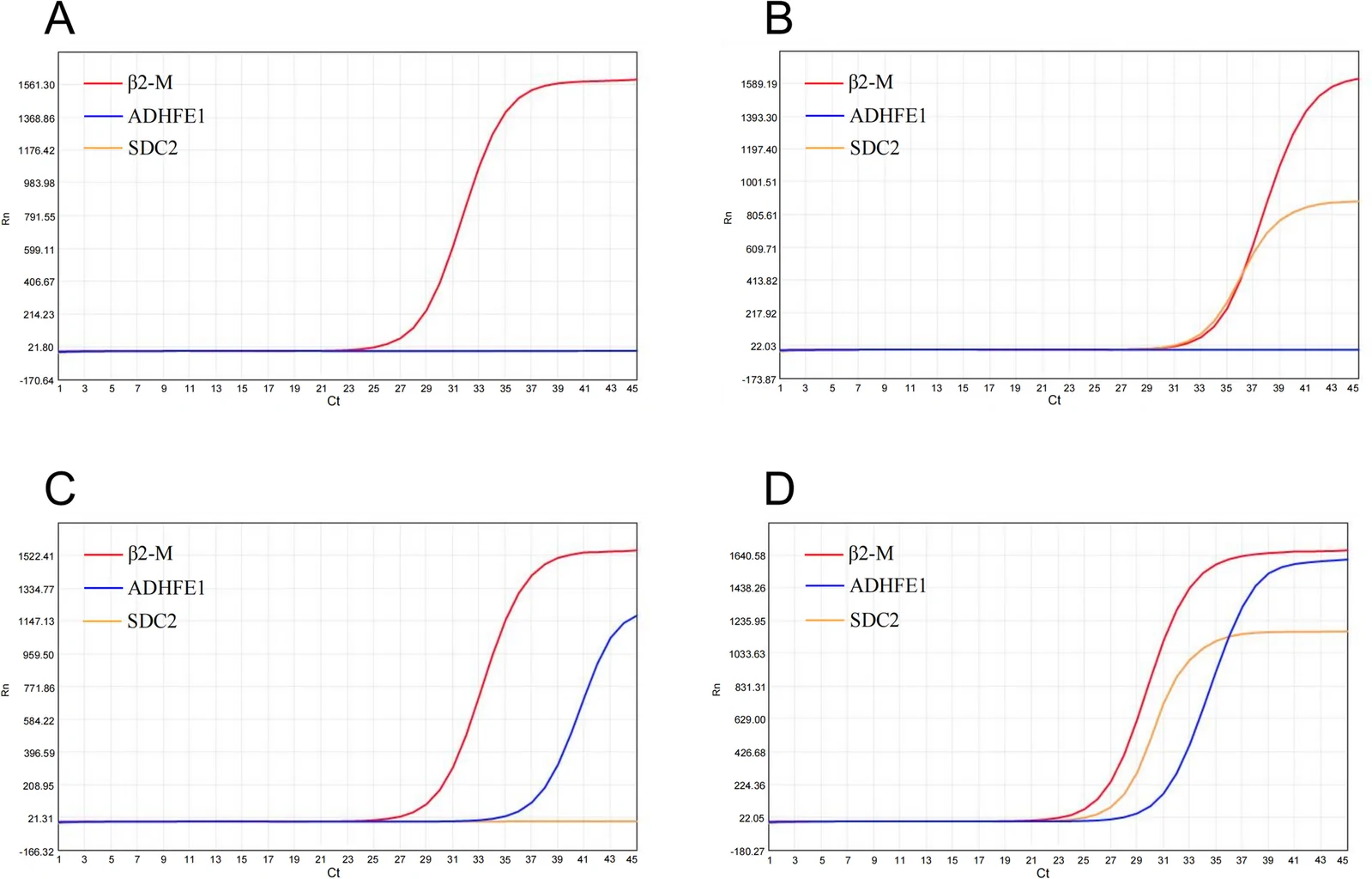
The MSRE-based assay for SDC2 and ADHFE1 Detection. **A** Amplification curve of HC fecal DNA samples. **B** Amplification curve of CRC fecal DNA samples: SDC2 positive. **C** Amplification curve of CRC fecal DNA samples: ADHFE1 positive. **D** Amplification curve of CRC fecal DNA samples: SDC2 + ADHFE1 positive

As depicted in Fig. 2A, fecal DNA from HC exhibited normal amplification of the internal reference gene β2-M after MSRE treatment, indicating the absence of enzymatic cleavage at this locus. In contrast, target biomarkers SDC2 and ADHFE1 showed no amplification signals, demonstrating complete digestion of unmethylated sequences in non-CRC controls. Conversely, fecal DNA from samples confirmed to be methylated at SDC2 and ADHFE1 by bisulfite sequencing maintained amplification for all β2-M, SDC2 and ADHFE1 after enzyme treatment, suggesting methylation-mediated resistance and protection to restriction digestion at the SDC2 and ADHFE1 loci (Fig. 2B, C). Figure 2D integrates these results, showing co-amplification of β2-M, SDC2 and ADHFE1 post-enzyme treatment in one CRC sample, validating the assay’s specificity for concurrent methylation abnormalities in CRC-associated genes. These data demonstrated robust technical specificity for detecting DNA methylation by the MSRE-based assay.

### Digestion efficiency of MSRE enzyme mixture

To optimize digestion efficiency, we compared an equimolar mixture of HpaII and HhaI with individual enzymes (Table 3). The internal control β2-M displayed stable Ct values across all conditions due to lacking recognition sites for these enzymes tested. However, individual enzymes (HpaII or HhaI alone) failed to fully digest unmethylated DNA in negative controls, yielding false-positive amplification signals. In contrast, the HpaII/HhaI mixture completely eliminated non-specific amplification in negative samples (undetermined Ct values), confirming synergistic enzymatic activity is essential for methylation-specific discrimination.

**Table 3:** Digestion efficiency of MSREs (negative control)

Restriction Enzyme Addition	Ct (β2-M)	Ct (ADHFE1)	Ct (SDC2)
Nuclease-free water (Control)	28.66	29.73	29.75
HpaII	28.51	34.74	38.12
HhaI	28.77	38.14	40.61
HpaII + HhaI (1:1 mixture)	28.62	Undetermined	Undetermined

### Clinical characteristics of all participants

A total of 274 participants were enrolled in this study, comprising 162 males (59.5%) and 112 females (40.5%). The participants were categorized into six groups based on colonoscopy and histopathological findings: CRC (*n* = 117), AA (*n* = 22), polyps (*n* = 30), IDC (*n* = 39), OGDC (*n* = 24) and HC (*n* = 42). Sex distribution showed no significant differences across groups. Comprehensive clinicopathological characteristics are in Table 4.

**Table 4:** Clinical characteristics of the study subjects

Characteristic	ALL	CRC	Advanced adenomas	Polyps	Intestinal disease control (IDC)	Other gastrointestinal disease control (OGDC)	HC	*P*
Number	274	117	22	30	39	24	42	
Age, years	56 (42–66)	64 (56–69)	62.5 (52-71.5)	56 (45–65)	32 (23–50)	54.5 (42.25–66.5)	43 (32.25–51.75)	< 0.01
Gender								0.286
Male	162 (59.5%)	71 (60.7%)	14 (63.6%)	16(53.3%)	26 (66.7%)	9 (37.5%)	26 (61.9%)	
female	112 (40.5%)	46 (39.3%)	8(36.4%)	14(46.7%)	13 (33.3%)	15 (62.5%)	16 (38.1%)	
TNM stage, *n* (%)								
Ⅰ	/	8 (6.8%)	/	/	/	/	/	
Ⅱ	/	42 (35.9%)	/	/	/	/	/	
Ⅲ	/	40 (34.2%)	/	/	/	/	/	
Ⅳ	/	27 (23.1%)	/	/	/	/	/	
Differentiation Degree								
Well and Moderate	/	83 (70.9%)	/	/	/	/	/	
Poorly	/	25 (21.4%)	/	/	/	/	/	
miss	/	9 (7.7%)	/	/	/	/	/	

### Comparative analysis of SDC2 and ADHFE1 methylation status in clinical groups

The methylation status of ADHFE1 and SDC2 across clinical groups is summarized in Table 5. The CRC group exhibited significantly higher methylation positivity rates for both SDC2(65.0%) and ADHFE1(64.1%) methylation compared to all control groups: AA (SDC2: 27.3%; ADHFE1: 22.7%), polyps (both genes: 6.7%), IDC (SDC2: 15.4%; ADHFE1: 7.7%), ODGC (SDC2: 0%; ADHFE1: 4.2%) and HC (SDC2: 0%; ADHFE1: 2.38%). Notably, significant differences in hypermethylation rates were also observed between the AA group and non-neoplastic subgroups (polyps, IDC, ODGC and HC), suggesting progressive methylation alterations spectrum during colorectal carcinogenesis.

**Table 5:** Methylation results of SDC2 and ADHFE1 in all participants

Characteristic	*n*	SDC2 methylation		ADHFE1 methylation		SDC2 + ADHFE1 methylation	
		Positive	Negative	Positive	Negative	Positive	Negative
CRC	117	76 (65.0%)	41 (35.0%)	75 (64.1%)	42 (35.9%)	97 (82.9%)	20 (17.1%)
Advanced adenomas	22	6 (27.3%)	16 (72.7%)	5 (22.7%)	17 (77.3%)	9 (40.9%)	13 (59.1%)
Polyps	30	2 (6.7%)	28 (93.3%)	2 (6.7%)	28 (93.3%)	2 (6.7%)	28 (93.3%)
Intestinal disease control	39	6 (15.4%)	33 (84.6%)	3 (7.7%)	36 (92.3%)	7 (17.9%)	32 (82.1%)
Other gastrointestinal disease control	24	0 (0.0%)	24 (100%)	1 (4.2%)	23 (95.8%)	1 (4.2%)	23 (95.8%)
HC	42	0(0%)	42(100%)	1(2.38%)	41(97.62%)	1(2.38%)	41(97.62%)
*P*			< 0.001		< 0.01		< 0.01
Stage Ⅰ+Ⅱ CRC	50	33 (66.0%)	17 (34%)	33 (66.0%)	17 (34%)	42 (84.0%)	8 (16.0%)
StageⅢ+ⅣCRC	67	43 (64.2%)	24 (35.8%)	42 (62.7%)	25 (37.3%)	55 (82.1%)	12 (17.9%)
*P*			0.838		0.712		0.57
Differentiation Degree*	108	72(66.67%)	36(33.33%)	69 (63.89%)	39 (36.11%)	91 (84.26%)	17 (15.74%)
Well and Moderate	83	54 (65.1%)	29 (34.9%)	52 (62.7%)	31 (37.3%)	69 (83.1%)	14 (16.9%)
Poorly	25	18 (72.0%)	7 (28.0%)	17 (68.0%)	8 (32.0%)	22 (88.0%)	3 (12.0%)
*P*			0.519		0.625		0.785

* Differentiation degree data available for 108/117 cases

Analysis of methylation status across CRC clinical subgroups revealed no significant differences (Table 5). For early-stage (I + II) versus advanced-stage (III + IV) CRC, positivity rates were comparable: SDC2 (66.0% vs. 64.2%, *P* = 0.838), ADHFE1 (66.0% vs. 62.7%, *P* = 0.712), and combined SDC2 + ADHFE1 (86.0% vs. 82.1%, *P* = 0.57). Similarly, tumor differentiation grade (well/moderate vs. poorly differentiated) showed no association with methylation positivity: SDC2 (65.1% vs. 72.0%, *P* = 0.519), ADHFE1 (62.7% vs. 68.0%, *P* = 0.625), and combined detection (83.1% vs. 88.0%, *P* = 0.785). These findings indicate that methylation alterations occur early in tumorigenesis and remain stable throughout CRC progression.

### Diagnostic value of SDC2 and ADHFE1 methylation in CRC detection

**Fig. 3:**
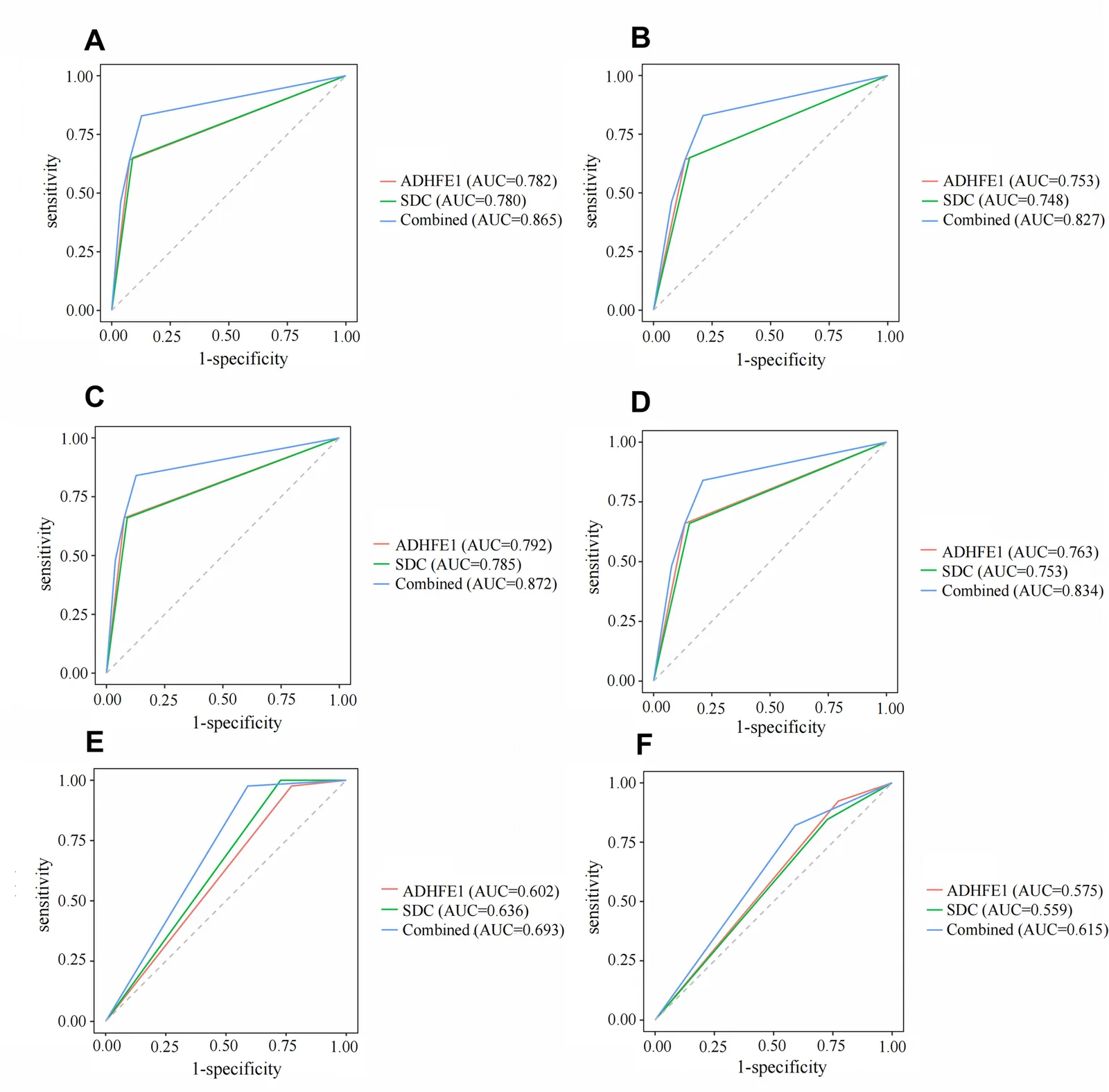
The diagnostic value of SDC2 and ADHFE1. **A** CRC vs. nCRC. **B** CRC vs. AA+Polyps. **C **Stage Ⅰ+Ⅱ CRC vs. nCRC. **D** StageⅠ+ⅡCRC vs. AA+Polyps. **E** AA vs. HC. **F** AA vs. IDC

Logistic regression analysis demonstrated that a positive combined ADHFE1/SDC2 methylation test was strongly associated with CRC diagnosis, with an unadjusted odds ratio (OR) of 33.22 (95% CI: 16.96–65.07, *P* < 0.001). This association remained robust after adjusting for age and sex, yielding an adjusted OR of 29.44 (95% CI: 14.34–60.41, *P* < 0.001)(Table 6). The diagnostic performance of methylated SDC2, ADHFE1 and their combined detection for CRC is summarized in Table 7; Fig. 3. When distinguishing CRC from non-CRC controls (AA+polyps + IDC+OGDC + HC), the dual-gene panel achieved significantly higher sensitivity (82.9%) compared to single gene test (ADHFE1:64.1%, SDC2:65%). Specificity remained robust for all tests (ADHFE1: 92.4%; SDC2: 91.1%; combined: 87.3%). Receiver operating characteristic (ROC) analysis revealed superior discriminative capacity for the combined assay, with an AUC of 0.865 (95% CI: 0.817–0.913), exceeding single-gene performance (ADHFE1 AUC: 0.782, 95% CI: 0.722–0.842; SDC2 AUC: 0.779, 95% CI: 0.719–0.839).

**Table 6:** Logistic regression for CRC

Variables	Model 1		Model 2	
	OR(95% CI)	*P*	OR(95% CI)	*P*
ADHFE1	21.58(10.72–43.43)	< 0.001	18.89(8.99–39.65)	< 0.001
SDC2	18.93(9.71–36.91)	< 0.001	16.55(8.15–33.62)	< 0.001
ADHFE1/SDC2	33.22(16.96–65.07)	< 0.001	29.44(14.34–60.41)	< 0.001

Model 1: None variables were adjusted

Model 2: gender and age were adjusted

**Table 7:** Diagnostic efficiency of SDC2 or ADHFE1 methylated for CRC

Group	Marker	AUC	AUC 95% CI	Sensitivity	Specificity
CRC vs. nCRC	ADHFE1	0.782	0.722–0.842	0.641	0.924
	SDC2	0.779	0.719–0.839	0.65	0.911
	ADHFE1/SDC2	0.865	0.817–0.913	0.829	0.873
CRC vs. AA+Polyps	ADHFE1	0.753	0.677–0.830	0.641	0.865
	SDC2	0.748	0.670–0.826	0.65	0.846
	ADHFE1/SDC2	0.827	0.758–0.896	0.829	0.788
StageⅠ+Ⅱ CRC vs. nCRC	ADHFE1	0.792	0.709–0.875	0.66	0.924
	SDC2	0.785	0.702–0.869	0.66	0.911
	ADHFE1/SDC2	0.872	0.807–0.937	0.84	0.873
Stage Ⅰ+ⅡCRC vs. AA+Polyps	ADHFE1	0.763	0.667–0.859	0.66	0.865
	SDC2	0.753	0.656–0.850	0.66	0.846
	ADHFE1/SDC2	0.834	0.752–0.917	0.84	0.788
AA vs. HC	ADHFE1	0.602	0.448–0.756	0.227	0.976
	SDC2	0.636	0.483–0.790	0.273	1
	ADHFE1/SDC2	0.693	0.544–0.842	0.409	0.976
AA vs. IDC	ADHFE1	0.575	0.420–0.730	0.227	0.923
	SDC2	0.559	0.406–0.713	0.273	0.846
	ADHFE1/SDC2	0.615	0.463–0.767	0.409	0.821

When differentiating CRC from AA and polyps, the combined assay maintained enhanced sensitivity (82.9%) relative to single-gene tests (ADHFE1: 64.1%; SDC2: 65.0%). Specificities were 86.5% (ADHFE1), 84.6% (SDC2), and 78.8% (combined). The dual-gene panel achieved an AUC of 0.827(95% CI: 0.758–0.896), outperforming individual markers (ADHFE1 AUC: 0.753, 95% CI: 0.677–0.830; SDC2 AUC: 0.748, 95% CI: 0.670–0.826).

For early-stage CRC detection (vs. non-CRC), the combined assay demonstrated further improved accuracy (AUC: 0.872, 95% CI: 0.807–0.937; sensitivity: 84.0%, specificity: 87.3%) surpassing single-gene assays (ADHFE1: AUC: 0.792; SDC2: AUC: 0.785). When distinguishing early-stage CRC from AA and polyps, performance remained high (AUC: 0.834, 95% CI: 0.752–0.917; sensitivity: 84.0%, specificity: 78.8%).

However, the dual-gene panel showed limited efficacy in differentiating AA from HC (AUC: 0.693, sensitivity: 40.9%, specificity: 97.6%) and IDC(AUC: 0.615, sensitivity: 40.9%, specificity: 82.1%). Nevertheless, it outperformed single gene assays (HC: ADHFE1: AUC: 0.602; SDC2: AUC: 0.636; IDC: ADHFE1: AUC: 0.575; SDC2: AUC: 0.559).

## Discussion

Early diagnosis remains critical for reducing the incidence and mortality of CRC, yet conventional serum biomarkers exhibit low sensitivity for early-stage lesions [19–21]. For instance, CEA and CA19-9 demonstrated sensitivities of only 37% and 13%, respectively [21]. These findings underscore the need for more robust biomarkers to improve early detection strategies. Accumulating evidence highlights the pivotal role of DNA methylation in CRC tumorigenesis and progression [22, 23] and support stool-derived methylation biomarkers for early-stage CRC detection. In this study, the MSRE-based methylation detection assay demonstrated robust technical and clinical performance, suggesting its viability as an effective alternative to bisulfite-dependent methods for non-invasive CRC screening. This enzymatic specificity, driven by HpaII and HhaI activity targeting distinct CpG site, overcomes the inherent limitations of bisulfite-based approaches, such as DNA degradation and incomplete conversion bias, which typically reduce sensitivity in low-abundance fecal DNA [24, 25].

We systematically evaluated the diagnostic value of single-gene (SDC2 or ADHFE1) and dual-gene methylation analysis in CRC and precancerous lesions using fecal samples. The combined SDC2/ADHFE1 assay outperformed single gene assays, achieving a sensitivity of 82.9% (vs. 64.1% for ADHFE1 and 65.0% for SDC2 alone) and an AUC of 0.865 (95% CI: 0.817–0.913) for discriminating CRC from non-CRC groups. For early-stage CRC (Ⅰ+Ⅱ), the dual-gene panel further improved diagnostic accuracy (AUC: 0.872), highlighting its potential for high-risk population screening. Although the specificity of the combined SDC2/ADHFE1 was marginally lower than that of single-gene assays, its superior sensitivity may prioritize its use in screening scenarios to minimize false-negative risks.

SDC2, a transmembrane protein, has been implicated in the regulation of cell proliferation and adhesion [26]. The methylation status of SDC2 shows variable reported sensitivities, with 86.21% (25/29) for stage Ⅰ+Ⅱ CRC and 88.89% (24/27) for stage Ⅲ+Ⅳ CRC. Notably, SDC2 methylation levels exhibit a significant positive correlation with lesion severity [27]. However, conflicting evidence indicates that detection rates of SDC2 methylation do not vary significantly based on tumor differentiation or TNM stage, reporting sensitivities of 83.8% for CRC, 42.1% AA and 87.0% for stage Ⅰ+Ⅱ CRC [28], which were consistent with our observations.

Studies have reported that single methylation assays targeting distinct SDC2 regions (SDC2-A and SDC2-B) exhibit high diagnostic performance. The SDC2-A assay achieved a sensitivity of 85.29% and specificity of 96.15% for CRC detection, while the SDC2-B assay yielded a sensitivity of 83.33% and specificity of 97.69%. Notably, combining SDC2-A and SDC2-B methylation analyses improved sensitivity to 87.25% with maintained specificity (94.62%), demonstrating that dual-fragment co-detection enhances diagnostic accuracy [29]. Barták et al. [30] further identified SDC2 methylation as the plasma biomarker in a study of 47 CRC patients, achieving 87.2% sensitivity and 100% specificity (AUC: 0.930). However, the limited sample size underscores the need for larger validation studies to confirm the clinical utility of SDC2 methylation in blood-based CRC screening.

ADHFE1 hypermethylation regulates oncogenic processes such as cell cycle progression and angiogenesis [31]. While ADHFE1 is upregulated in gastric cancer [32], it is downregulated in CRC tissues, correlating with poor differentiation and advanced stages. Li et al. [18] reported that the sensitivity of ADHFE1 methylation for CRC detection was 81.9%. For distinguishing CRC from individuals with no evidence of disease, the combined SDC2/ADHFE1/PPP2R5C panel achieved an AUC of 0.930 with 84.8% sensitivity and 98.0% specificity, while ADHFE1 alone had the AUC of 0.893. In contrast, our study observed slightly lower AUC values of ADHFE1. which may reflect the inclusion of clinical controls (non-CRC and AA/polyps) rather than healthy subjects alone.

In our data set, no significant differences were noted in methylation positivity rates between stage Ⅰ+Ⅱ and stage Ⅲ+Ⅳ CRC or among differentiation grades. These findings suggest that methylation alterations in these genes likely occur during early tumorigenesis and remain stable throughout disease progression, supporting their potential as early diagnostic biomarkers for CRC. Importantly, the low methylation positivity rates in polyps and IDC indicate that the specificity of these markers is primarily associated with malignant transformation rather than benign or inflammatory processes.

This present study confirms that combined detection of SDC2 and ADHFE1 methylation serves as a robust predictor for CRC diagnosis. After adjusting for age and sex, the adjusted OR was 29.44. This exceptionally high OR value primarily stems from the assay’s high detection rate in CRC patients and its high specificity in non-CRC controls. However, the relatively wide CI suggests the need for further validation in larger, prospective cohort studies.

This study examined the methylation status of the SDC2 and ADHFE1 genes in fecal samples. The results showed that the detection rate of a single gene was 65.0% (for SDC2) and 64.1% (for ADHFE1) in the CRC group. When both genes were jointly tested, the positive rate increased to 82.9%, significantly higher than each individual indicator. Compared with previous histological studies, Oh et al. reported that the methylation positive rate of SDC2 in CRC tissues was as high as 97.8% [33]. However, the detection rate of fecal SDC2 methylation in this study was relatively low, which might be related to the limited amount of tumor-derived DNA in feces and tumor heterogeneity. Nevertheless, SDC2 methylation in feces can still effectively distinguish CRC patients from healthy controls. For precancerous lesions, Park et al. found in the intestinal lavage fluid that the positive rate of SDC2 methylation gradually increased from normal mucosa (11.1%) to hyperplastic polyps (28.6%), single adenoma (26.7%), and multiple adenomas (62.8%) [34]. In this study, the combined positive rate of advanced adenomas in fecal samples was 40.9%, and that in the polyp group was 6.7%, showing a similar upward trend, suggesting that fecal testing can also reflect the progression status of colorectal tumors. Regarding ADHFE1, Tae et al. [35]. and Hu et al. [13] confirmed in the CRC tissue that there was frequent methylation in the promoter region of ADHFE1. In this study, the detection rate of ADHFE1 in feces for CRC was 64.1%, which was comparable to that of SDC2, and the combined detection further improved the sensitivity. It is notable that in the intestinal disease control group (such as inflammatory bowel disease), the positive rate of combined methylation was 17.9%, slightly higher than that of the healthy control group (2.38%), suggesting that certain non-tumor intestinal diseases may also have low-level methylation signals. In addition to the epigenetic markers discussed in this study, the CT-based radiomics analysis also demonstrates potential in predicting KRAS/NRAS/BRAF mutations, providing another layer of non-invasive information support for the treatment of colorectal cancer and offering information for individualized targeted therapy [36].

Notably, while there were no significant differences detected in SDC2, ADHFE1, or combined SDC2 + ADHFE1 methylation positivity rates between early-stage (Ⅰ+Ⅱ) and advanced-stage (Ⅲ+Ⅳ) CRC, the early-stage group exhibited numerically higher methylation positivity rates across all assays conducted. This trend likely contributed to the marginally elevated AUC values for the combined panel when comparing of stage Ⅰ+Ⅱ CRC versus non-CRC as well as stage Ⅲ+Ⅳ CRC versus AA/polyps, this suggests enhanced discriminatory capacity for early-stage lesions despite the lack of statistical significance regarding differences in methylation rates.

This study has several strengths: (1) Development of a streamlined single-tube MSRE-PCR assay; (2) Inclusion of diverse pathological controls (AA, polyps, IDC, OGDC, HC); and (3) Consistent demonstration of superior CRC discrimination using the dual-gene panel. However, the assay showed limited efficacy in distinguishing AA from HC (AUC: 0.693) or IDC (AUC: 0.615), underscoring the need for complementary biomarkers to detect precancerous lesions.

### Limitations of the study

This study has several limitations. First, as a single-center, retrospective, cross-sectional investigation, it is inherently subject to selection bias. The patient cohort was drawn from a tertiary referral hospital, which may not fully represent the broader population undergoing routine screening, thus constraining the generalizability of our results to community or primary care settings. The exclusive focus on a Chinese population further limits the external validity of our findings across diverse ethnic groups. Second, regarding the precision of estimates, the diagnostic performance for key subgroups, particularly AA and polyps, is based on relatively small sample sizes (*n* = 22 and *n* = 30, respectively). This is reflected in the wider confidence intervals for the corresponding AUC estimates, indicating substantial statistical uncertainty. Future studies with larger, specifically enriched cohorts for these precursor lesions are required to obtain more precise estimates. Third, while laboratory analyses were performed blinded, the retrospective use of clinical data carries a risk of missing variables. Although we adjusted for age and sex in supplementary analyses, residual confounding by unmeasured factors (e.g., detailed lifestyle, comorbidities, or genetic background) could influence the observed associations. The direction of this potential bias is difficult to quantify but could lead to either an over- or underestimation of the true diagnostic accuracy. Fourth, a significant data limitation is the unexamined prognostic relevance of SDC2/ADHFE1 methylation status to long-term clinical outcomes, such as overall survival or response to therapy. Our study was not designed to address this important question. Finally, the absence of multi-center or external validation means that the performance of our MSRE-based assay, especially its operational robustness and reproducibility across different laboratories and patient populations, remains to be confirmed. Future work should prioritize expanding sample sizes, integrating additional promising epigenetic markers (NDRG4 and BMP3), and conducting rigorous multi-center trials to optimize and validate the diagnostic model before clinical implementation.

## Conclusion

Overall, SDC2 combined with ADHFE1 methylation test significantly improves the diagnostic sensitivity and differential efficacy of CRC, and its non-invasive nature are suitable as a complementary tool for colonoscopy screening. These findings underscore the superior efficacy of multi-gene methylation panels over single-marker tests, offering a promising strategy to improve early detection and reduce CRC-related mortality.

## Supplementary Information

Supplementary Material 1.

Supplementary Material 2.

## Data Availability

All data generated or analysed during this study are included in this published article.

## Abbreviations

MSRE: Methylation-sensitive restriction endonuclease
CRC: Colorectal cancer
SDC2: Syndecan-2
ADHFE1: Alcohol dehydrogenase iron containing 1
AUC: Area under the curve
ROC: Receiver operating characteristic curve
CI: Confidence interval
Ct: Threshold cycle value
AA: Advanced adenoma
IDC: Intestinal disease control
OGDC: Other gastrointestinal disease control
HC: Healthy control
FOBT: Fecal occult blood test
FIT: Fecal immunochemical test
β2-M: β2-microglobulin
