# Supplementary material for: Methylation-sensitive restriction endonuclease-based amplification assay for detecting DNA methylation in colorectal cancer: a cross-sectional study
Source: BMC Gastroenterol. 2026 Aug 3;26:713. doi: 10.1186/s12876-026-05034-7 (PMC13721474; doi:10.1186/s12876-026-05034-7)
Supplement: Supplementary file 1 [file 12876_2026_5034_MOESM1_ESM.docx]

Samples with positive ADHFE1 gene expression：

Samples with positive SDC2 gene expression：

Samples with negative ADHFE1 gene expression：

Samples with negative SDC2 gene expression：
