# Supplementary material for: Methylation-sensitive restriction endonuclease-based amplification assay for detecting DNA methylation in colorectal cancer: a cross-sectional study
Source: BMC Gastroenterol. 2026 Aug 3;26:713. doi: 10.1186/s12876-026-05034-7 (PMC13721474; doi:10.1186/s12876-026-05034-7)
Supplement: Supplementary file 2 [file 12876_2026_5034_MOESM2_ESM.doc]

STROBE Statement—checklist of items that should be included in reports of observational studies

|  | Item No | Recommendation | Response from authors |
| --- | --- | --- | --- |
| **Title and abstract** | 1 | (*a*) Indicate the study’s design with a commonly used term in the title or the abstract | Addressed (see Title and Abstract) |
|  |  | (*b*) Provide in the abstract an informative and balanced summary of what was done and what was found | Addressed (see Abstract) |
| Introduction | | |  |
| Background/rationale | 2 | Explain the scientific background and rationale for the investigation being reported | Addressed (see Introduction) |
| Objectives | 3 | State specific objectives, including any prespecified hypotheses | Addressed (see Introduction) |
| Methods | | |  |
| Study design | 4 | Present key elements of study design early in the paper | Addressed (see "Patient Cohort" section in the "Materials and Methods") |
| Setting | 5 | Describe the setting, locations, and relevant dates, including periods of recruitment, exposure, follow-up, and data collection | Addressed (see "Patient Cohort" section in the "Materials and Methods") |
| Participants | 6 | (*a*) *Cohort study*—Give the eligibility criteria, and the sources and methods of selection of participants. Describe methods of follow-up  *Case-control study*—Give the eligibility criteria, and the sources and methods of case ascertainment and control selection. Give the rationale for the choice of cases and controls  *Cross-sectional study*—Give the eligibility criteria, and the sources and methods of selection of participants | Addressed (see "Patient Cohort" section in the "Materials and Methods") |
|  |  | (*b*) *Cohort study*—For matched studies, give matching criteria and number of exposed and unexposed  *Case-control study*—For matched studies, give matching criteria and the number of controls per case | Not applicable.This study was not cohort study or case-control. |
| Variables | 7 | Clearly define all outcomes, exposures, predictors, potential confounders, and effect modifiers. Give diagnostic criteria, if applicable | Addressed (see "Patient Cohort" and "Assessment and Control of Potential Biases" section in the "Materials and Methods") |
| Data sources/ measurement | 8* | For each variable of interest, give sources of data and details of methods of assessment (measurement). Describe comparability of assessment methods if there is more than one group | Addressed (see "Data Sources and Measurement" section in the "Materials and Methods") |
| Bias | 9 | Describe any efforts to address potential sources of bias | Addressed (see "Assessment and Control of Potential Biases" section in the "Materials and Methods") |
| Study size | 10 | Explain how the study size was arrived at | Addressed (see "Patient Cohort" section in the "Materials and Methods") |
| Quantitative variables | 11 | Explain how quantitative variables were handled in the analyses. If applicable, describe which groupings were chosen and why | Addressed (see "Statistical analysis" section in the "Materials and Methods") |
| Statistical methods | 12 | (*a*) Describe all statistical methods, including those used to control for confounding | Addressed (see "Statistical analysis" section in the "Materials and Methods") |
|  |  | (*b*) Describe any methods used to examine subgroups and interactions | Addressed (see "Statistical analysis" section in the "Materials and Methods") |
|  |  | (*c*) Explain how missing data were addressed | In the subgroup analysis based on tumor differentiation degree (Table 5), 9 cases with missing differentiation data were excluded, and the analysis was conducted based on the remaining 108 cases. |
|  |  | (*d*) *Cohort study*—If applicable, explain how loss to follow-up was addressed  *Case-control study*—If applicable, explain how matching of cases and controls was addressed  *Cross-sectional study*—If applicable, describe analytical methods taking account of sampling strategy | We did not employ a sampling strategy. Instead, we consecutively enrolled all eligible cases during a defined period. This consecutive enrollment approach aimed to minimize selection bias. |
|  |  | (*e*) Describe any sensitivity analyses | In this study, all key diagnostic outcomes (positive/negative) were determined by a pre-specified, objective ΔCt threshold with no ambiguous cases, and there were no missing data in the primary analysis. Therefore, the standard analysis already represents. |

Continued on next page

| Results | | |  |
| --- | --- | --- | --- |
| Participants | 13* | (a) Report numbers of individuals at each stage of study—eg numbers potentially eligible, examined for eligibility, confirmed eligible, included in the study, completing follow-up, and analysed | Addressed (see "Clinical characteristics of all participants" section in the "Results" ) |
|  |  | (b) Give reasons for non-participation at each stage | Addressed (see Figure 1 STROBE flow diagram of participant selection and grouping. ) |
|  |  | (a) Give characteristics of study participants (eg demographic, clinical, social) and information on exposures and potential confounders | Addressed (see "Clinical characteristics of all participants" section in the "Results" ) |
|  |  | (b) Indicate number of participants with missing data for each variable of interest | Addressed (see Table 5) |
|  |  | (c) *Cohort study*—Summarise follow-up time (eg, average and total amount) | Not applicable.This study was not cohort study or case-control. |
|  |  | *Cohort study*—Report numbers of outcome events or summary measures over time | Not applicable.This study was not cohort study or case-control. |
|  |  | *Case-control study—*Report numbers in each exposure category, or summary measures of exposure | Not applicable.This study was not cohort study or case-control. |
|  |  | *Cross-sectional study—*Report numbers of outcome events or summary measures | Addressed (see "Clinical characteristics of all participants" section in the "Results" ) |
|  |  | (*a*) Give unadjusted estimates and, if applicable, confounder-adjusted estimates and their precision (eg, 95% confidence interval). Make clear which confounders were adjusted for and why they were included | Addressed (see Table 6) |
|  |  | (*b*) Report category boundaries when continuous variables were categorized | Addressed (see "DNA isolation and Methylation detection of SDC2 and AHDFE1 genes " section in the "Materials and Methods").  We have clearly stated the specific thresholds used to categorize the continuous ΔCt values into "positive" and "negative" (SDC2: ΔCt ≤ 9; ADHFE1: ΔCt ≤ 10). |
|  |  | (*c*) If relevant, consider translating estimates of relative risk into absolute risk for a meaningful time period | Based on the cross-sectional diagnostic design of this study, which aimed to evaluate the performance of the assay rather than to perform long-term risk prediction, the translation of OR into absolute risk estimates was not conducted. |
|  |  | Report other analyses done—eg analyses of subgroups and interactions, and sensitivity analyses | Addressed (see Table 5) |
| Discussion |  |  |  |
| Key results | | | 18 |
| LimitationsSummarise key results with reference to study objectivesAddressed (see Discussion) | 19 | Discuss limitations of the study, taking into account sources of potential bias or imprecision. Discuss both direction and magnitude of any potential bias | Addressed (see Limitations of the Study) |
| Interpretation | 20 | Give a cautious overall interpretation of results considering objectives, limitations, multiplicity of analyses, results from similar studies, and other relevant evidence | Addressed (see Discussion) |
| Generalisability | 21 | Discuss the generalisability (external validity) of the study results | Addressed (see Discussion) |
| Other information |  |  |  |
| Funding | | | 22 |
| Give the source of funding and the role of the funders for the present study and, if applicable, for the original study on which the present article is basedAddressed (see Funding) |  |  |  |

*Give information separately for cases and controls in case-control studies and, if applicable, for exposed and unexposed groups in cohort and cross-sectional studies.

**Note:** An Explanation and Elaboration article discusses each checklist item and gives methodological background and published examples of transparent reporting. The STROBE checklist is best used in conjunction with this article (freely available on the Web sites of PLoS Medicine at http://www.plosmedicine.org/, Annals of Internal Medicine at http://www.annals.org/, and Epidemiology at http://www.epidem.com/). Information on the STROBE Initiative is available at www.strobe-statement.org.
